# Non-fermentative gram-negative bloodstream infection in northern Italy: a multicenter cohort study

**DOI:** 10.1186/s12879-021-06496-8

**Published:** 2021-08-12

**Authors:** Renato Pascale, Silvia Corcione, Linda Bussini, Livia Pancaldi, Daniele Roberto Giacobbe, Simone Ambretti, Tommaso Lupia, Cristina Costa, Anna Marchese, Francesco Giuseppe De Rosa, Matteo Bassetti, Claudio Viscoli, Michele Bartoletti, Maddalena Giannella, Pierluigi Viale

**Affiliations:** 1 Infectious Diseases Unit, Department of Medical and Surgical Sciences, IRCCS Azienda Ospedaliero-Universitaria di Bologna, Via Massarenti 11, 40137 Bologna, Italy; 2 Department of Medical Sciences, Infectious Diseases, University of Turin, Turin, Italy; 3 Infectious Diseases Unit, Ospedale Policlinico San Martino-IRCCS, Genoa, Italy; 4 Department of Health Sciences (DISSAL), University of Genoa, Genoa, Italy; 5 Operative Unit of Clinical Microbiology, Policlinico Sant’Orsola Malpighi, University of Bologna, Bologna, Italy; 6 Department of Public Health and Pediatrics, Laboratory of Microbiology and Virology, Città della Salute e della Scienza Hospital, University of Turin, Turin, Italy; 7 Department of Surgical Sciences and Integrated Diagnostics (DISC), University of Genoa, Genoa, Italy; 8 Microbiology Unit, Ospedale Policlinico San Martino-IRCCS, Genoa, Italy

**Keywords:** Bloodstream infection, Non-fermentative gram-negative bacteria, Multidrug resistance, Difficult to treat resistance, Therapeutic management, 30-Day mortality

## Abstract

**Background:**

The management of non-fermentative gram-negative bloodstream infection (NFGN-BSI) offers numerous challenges. In this study the aim is to analyse a large cohort of patients with NFGN-BSI recruited in the northern Italy to describe epidemiology, etiological and susceptibility pattern, therapeutic management and outcome.

**Methods:**

Multicentre retrospective cohort study of patients hospitalised at three large teaching hospitals in northern Italy in a fourth year period.

**Results:**

355 BSI episodes were analyzed, due to *P. aeruginosa* (72.7%), *A. baumannii* (16.6%), and *Stenotrophomonas maltophilia* (10.7%). Overall, 21.4% of isolates were defined as DTR, highest rate among *A. baumannii* (64.4%). All-cause 30-day mortality rate was 17.5%. Rates of XDR or DTR *A. baumannii* isolation were significantly higher in non-surviving patients. Independent risk factors for 30-day mortality were: age (HR 1.03, 95%CI 1.00–1.04, p = 0.003), septic shock (HR 2.84, 95%CI 1.67–4.82, p < 0.001) and BSI due to *Acinetobacter baumannii* (HR 2.23, 95%CI 1.27–3.94, p = 0.005).

**Conclusion:**

The overall prevalence of DTR was high in the NFGN BSI cohort analyzied, mainly among *Acinetobacter baumannii* episodes (64.4%). *Acinetobacter baumannii* is showed to be an independent predictor of mortality. These evidences marked the urgent need of new therapeutic options against this pathogen.

*Trial registration number:* 79/2017/O/OssN. Approved: March14th, 2017.

**Supplementary Information:**

The online version contains supplementary material available at 10.1186/s12879-021-06496-8.

## Background

The management of non-fermentative gram-negative bloodstream infection (NFGN-BSI) offers numerous challenges. Indeed, there are several clinical and microbiological issues that may contribute to its high morbidity and mortality. NFGN-BSI is usually diagnosed in people with severe underlying conditions, critically ill and/or immunocompromised patients [1–3]. Isolates are generally resistant, or prone to acquire resistance, to first-line antibiotics resulting in a high rate of initial inappropriate therapy and/or in the use of less effective and more toxic drugs. To counteract these findings, antibiotic combination regimens are frequently employed with controversial results in terms of efficacy, toxicity and collateral environmental damage [3, 4].

The knowledge of local epidemiology, etiological distribution in terms of causative agents and antibiotic resistance, therapeutic approach and factors associated with poor outcome are useful to guide infection control and antimicrobial stewardship policies and to inform clinicians regarding the best treatment approach [4].

With this premise, we analysed a large cohort of patients with NFGN-BSI recruited in three regions of northern Italy to describe the current epidemiology, etiological and susceptibility pattern distribution, therapeutic management and outcome.

## Methods

### Study design and setting

Multicentre retrospective cohort study of patients hospitalised at three large teaching hospitals in northern Italy: (i) Sant’Orsola Malpighi Hospital, Bologna; (ii) City of Health and Sciences, Molinette Hospital, Turin; (iii) San Martino Hospital, Genoa. The study period was from January 1st 2013 to December 31st 2016. Patients were identified through the records of the Microbiology Laboratory of each hospital.

Data source was the clinical charts and hospital records, reviewed until 90 days after the index blood cultures (BCs). Study variables were collected using a case report form. Data accuracy was assessed by a senior investigator. In addition, the numbers of patient days per year were recorded to assess the incidence of NFGN-BSI in the participating hospitals during the study period.

The study was approved by the institutional Ethics Committee of coordinating center, Sant’Orsola Malpighi Hospital (Comitato Etico di Area Vasta Emilia Centro—CE-AVEC79/2017/O/OssN). According to local rules, due to the retrospective nature of the study, the acquisition of consent by the interested parties was not envisaged in case of organizational impossibility. The collection of informed consent was obtained in all cases in which it was possible to provide adequate information to the patients. Patients were aware that samples could be used in research and that data could be published. Data were collected anonymously.

The antibiotic treatment, both empirical and definitive, was selected according to clinical judgment and not dictated by study protocol. As for polymyxin based therapy, colistin is the only drug available in Italy.

### Participants

All adult (≥ 18 years) patients diagnosed with NFGN-BSI were included in the study. NFGN-BSI was defined as one or more positive BCs obtained from a patient suspected of having infection. Patients were considered only once at the time of first episode (index BCs).

Exclusion criteria included: (i) polymicrobial BSI, defined as growth of more than one micro-organism, excluding potential contaminants (i.e. coagulase-negative staphylococci, *Corynebacterium* spp., *Propionibacterium* spp.); (ii) clinical data not available.

### Variables and definitions

The primary outcome was all-cause mortality within 30 days after index BCs [5]. The predictor variables included age and sex. The Charlson comorbidity index was used to asses underlying disease [6]. Immunosuppression included: neutropenia (neutrophil count < 500/mm^3^), solid organ transplantation, hematopoietic stem cell transplantation, corticosteroid therapy (at a dosage higher then or equivalent to prednisone 16 mg/day ≥ 15 days), uncontrolled human immunodeficiency virus (HIV) infection (< 200 CD4/mm^3^).

BSI was classified into nosocomial, healthcare-associated and community acquired using Friedman’s criteria [7]. Clinical severity at infection onset was assessed according to Sequential Organ Failure Assessment (SOFA) score and septic shock criteria [8]. BSI sources were established according to Centers for Disease Control and Prevention (CDC) criteria [9]. BSI was considered as primary in case of unrecognized source. BSI was defined as complicated when the infection source was not fully removable.

The susceptibility pattern of isolates was classified according to Magiorakos et al. criteria [10] as multidrug resistance (MDR) as nonsusceptibility to ≥ 1 agent in ≥ 3 antimicrobial categories; extensive drug resistance (XDR) as susceptibility limited to ≤ 2 categories; and pan-drug resistance (PDR) as nonsusceptibility to all agents in all antimicrobial categories.

In addition, CDC surveillance definitions were used to assess susceptibility to carbapenems, extended-spectrum cephalosporins (ESC) and fluoroquinolones (FQ) (https://gis.cdc.gov/grasp/PSA/Downloads/AR-PhenotypeDefinitions.pdf). Moreover susceptibility to betalactam/betalactamase inhibitors (BL/BLI) and colistin was determined according to European Committee for Antimicrobial Susceptibility Testing (EUCAST) criteria.

The new definition of “difficult to treat resistance” (DTR) was also assessed as reported elsewhere [11, 12]. Empirical therapy was defined as antibiotics administered before the susceptibility report was available. Appropriate empirical therapy was defined as a therapy contained at least one in vitro active drug (according to the susceptibility pattern of the isolate) administered within 24 h after drawing index BCs. Inappropriate empirical therapy was defined as inactive antibiotic administration or a delayed antibiotic therapy. Antibiotic treatment administered according to susceptibility results was considered as definitive antibiotic therapy. Antibiotic regimens including more than one anti-gram-negative agents, irrespective of their in vitro activity against the BSI isolate, during more than 50% of treatment duration were defined as “combination regimen”. Antibiotic therapy including at least two drugs showing in vitro activity against the BSI isolate was labelled as “2-in vitro active combination regimen”. Duration of antibiotic treatment was defined as the number of consecutive days during which the patient received an appropriate antibiotic regimen. Source control was defined as the removal of the infection source within 7 days of index BCs, including the performance of non-surgical or surgical procedures to treat an obstructive focus or abscess at any site including, among others, the urinary tract, biliary tract and surgical site, and the removal of any device deemed as the source of BSI.

### Microbiology

BCs were incubated using the BACTEC FX Automated Blood Culture System (Becton Dickinson, Franklin Lakes, NJ). All positive BCs were processed with Maldi Biotyper MALDI-TOF system (Bruker Daltonics, Bremen, Germany) for rapid and reliable species identification of microorganisms. Antimicrobial susceptibility testing was performed using the Vitek 2 automated system (bioMerieux, Marcy l’Etoile, France) in two hospitals (Bologna and Genova) and the MicroScan system in the remaining hospital (Torino). The minimum inhibitory concentrations (MICs) were interpreted using EUCAST clinical breakpoints for all tested antibiotics.

### Statistical analysis

For the descriptive analysis, categorical variables were presented as absolute numbers and their relative frequencies. Continuous variables were presented as the mean and standard deviation if normally distributed or as the median and interquartile range (IQR) if non-normally distributed.

Univariate and multivariate analysis were used to assess risk factors for all-cause 30-day mortality. Categorical variables were compared using χ^2^ or Fisher exact test when appropriate. Continuous variables were compared using the Mann–Whitney U-test. Significant and clinically relevant covariates identified in univariate analysis were introduced into a multivariable Cox regression survival model after verifying for proportional hazards and collinearity. Significance was considered for p < 0.05. All the analysis were performed using SPSS software.

## Results

**Fig. 1:**
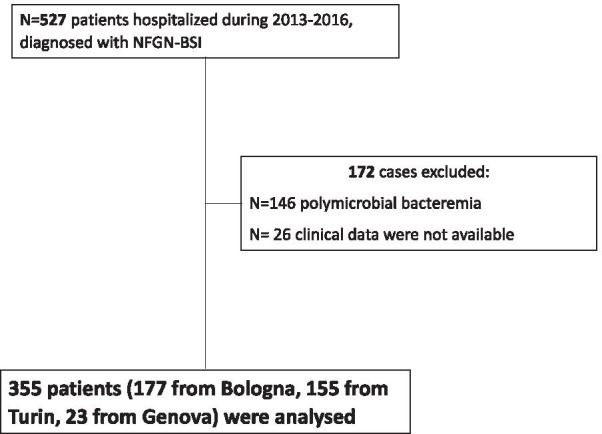
Flow diagram of study population

Over the study period, 527 patients were diagnosed with NFGN-BSI. Of them, 172 were excluded: 146 patients had polymicrobial bacteremia, and for the remaining 26 patients clinical data were not available. Thus, 355 patients (177 from Bologna, 155 from Turin and 23 from Genova) were analysed (Fig. 1).

**Fig. 2:**
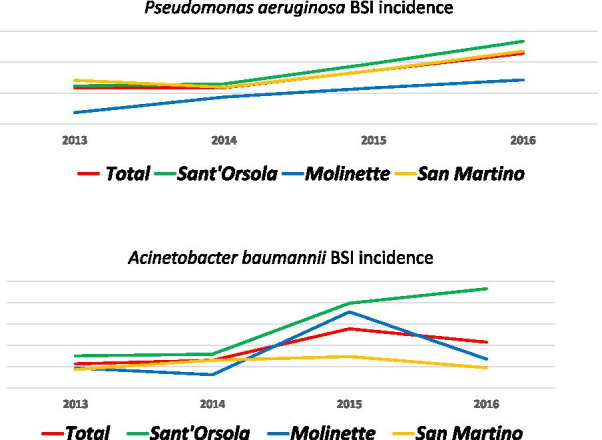
Overall incidence of *P. aeruginosa* and *A. baumannii* BSI per 1000 patient days between 2013 and 2016 years

The overall incidence of *Pseudomonas aeruginosa* BSI per 1000 patient days was 0.12, 0.12, 0.17, and 0.23 in 2013, 2014, 2015 and 2016, respectively. It was similar between participating hospitals. The overall incidence of *Acinetobacter baumannii* BSI per 1000 patient days was 0.02, 0.03, 0.06, and 0.04 in 2013, 2014, 2015 and 2016, respectively. It was similar between participating hospitals. Data shown in Fig. 2.

**Fig. 3:**
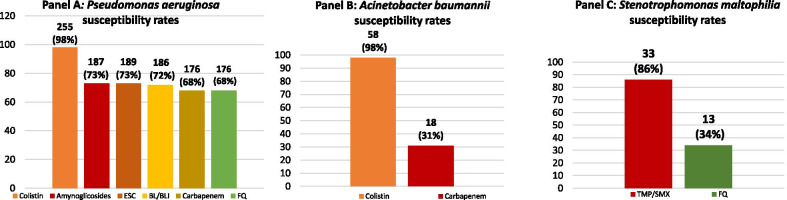
Susceptibility rates to individual antibiotic categories for *Pseudomonas aeruginosa*, *Acinetobacter baumannii* and *Stenotrophomonas malptophilia.* *BL/BLI* betalactam/betalactamase inhibitor, *ESC* extended-spectrum cephalosporin, *FQ* fluoroquinolone resistance

Etiological distribution and susceptibility patterns are shown in Table 1. Most episodes were due to *P. aeruginosa* (n = 258, 72.7%), followed by *A. baumannii* (n = 59, 16.6%), and *Stenotrophomonas maltophilia* (n = 38, 10.7%). Overall, 21.4% of isolates were defined as DTR with highest rate among *A. baumannii* (64.4%). Susceptibility rates to individual antibiotic categories are shown in Fig. 3. Both *P. aeruginosa* and *A. baumannii* maintained high rate susceptibility to colistin.

**Table. 1:** Causative agents and their susceptibility patterns of monomicrobial NFGN-BSI during 2013–2016 in three tertiary teaching hospitals from northern Italy

	N	MDR	XDR	PDR	CR	DTR
*Pseudomonas aeruginosa*	258	15 (5.8)	53 (20.5)	/	82 (31.8)	35 (13.6)
*Acinetobacter baumannii*	59	0	37 (62.7)	1(1.7)	41 (69.7)	38 (64.4)
*Stenotrophomonas maltophilia*	38	/	/	/	/	2 (5.3)

Resistance categories (MDR, XDR, PDR) were mutually exclusive while antibiotic class resistances (CR) and new definition (DTR) were not

*CR* carbapenem resistance, *DTR* difficult-to-treat resistance, *MDR* multidrug-resistance, *PDR* pandrug-resistance, *XDR* extensively drug-resistance

The general characteristics of study population are shown in Table 2. Overall, 65.6% of patients were male, the median age was 67 (IQR 55–79) years, the median Charlson index was 5.7 (IQR 3.6–7.4), and 24.5% were immunocompromised. Most patients were hospitalized at a medical ward at BSI onset (59.2%), and the majority of episodes were hospital acquired (70.1%). Infection source was not identified in 183 (51.5%) cases, in the remaining cases the most common sources of NFGN-BSI were CVC (n = 102) and lower respiratory tract (n = 49).

**Table. 2:** Univariate analysis of risk factors for all-cause 30-day mortality

	TotalN = 355 (%)	SurvivorsN = 293 (%)	Non-survivorsN = 62 (%)	*p*
*Demographics*				
Age (years) (median, IQR)	67 (55–79)	67 (54–78)	73 (61–84)	0.016
Male sex	233 (65.6)	194 (66.2)	39 (62.9)	0.66
*Comorbidities*				
Charlson index (median, IQR)	5.7 (3.6–7.4)	5.2 (3.5–7.1)	6.25 (4–9)	0.034
Immunosuppression	87 (24.5)	74 (25.3)	13 (21)	0.52
*Ward of admission*				0.22
Medical	210 (59.2)	176 (60.1)	34 (54.8)	
ICU	80 (22.5)	61 (20.8)	19 (30.6)	
Surgical	65 (18.3)	56 (19.1)	9 (14.5)	
*Site of BSI acquisition*				0.078
Community acquired	71 (20.0)	65 (22.2)	6 (9.7)	
Healthcare associated	35 (9.9)	27 (9.2)	8 (12.9)	
Hospital acquired	249 (70.1)	201 (68.6)	48 (77.4)	
CRE carrier at BSI onset	44 (12.1)	35 (11.9)	9 (14.5)	0.462
*Clinical severity at BSI onset*				
SOFA (median, IQR)	3 (2–5)	3 (2–5)	4 (3–6)	0.005
Septic shock	59 (16.6)	38 (13)	21 (33.9)	0.001
*Source of BSI*				
Undefined	183 (51.5)	150 (51.2)	33 (53.2)	0.782
CVC related	102 (28.7)	86 (29.4)	16 (25.8)	0.645
Lower respiratory tract	49 (13.8)	39 (13.3)	10 (16.1)	0.686
Biliary tract	41 (11.5)	34 (11.6)	7 (11.3)	1
Urinary tract	31 (8.7)	27 (9.2)	4 (6.5)	0.624
Intra-abdominal	18 (5.1)	16 (5.5)	2 (3.2)	0.551
Complicated BSI	38 (10.7)	27 (12.9)	11 (21.6)	0.125
*Etiology*				0.009
*Pseudomonas aeruginosa*	258 (72.7)	222 (75.8)	36 (58.1)	0.005
*Acinetobacter baumannii*	59 (16.6)	41 (14)	18 (29)	0.005
*Stenotrophomonas maltophilia*	38 (10.7)	30 (10.2)	8 (12.9)	0.651
Resistance phenotypes*				0.005
MDR	34 (9.6)	22 (8.4)	12 (22.2)	
XDR	58 (16.3)	46 (17.5)	12 (22.2)	
Antibiotic class resistance*				
ECR	95 (26.7)	77 (26.3)	18 (29)	0.752
BL/BLIR	78 (22)	55 (18.8)	23 (37.1)	0.002
CR	124 (34.9)	93 (31.7)	31 (50)	0.008
FQR	144 (40.6)	107 (36.5)	37 (59.7)	0.001
AminoglycosidesR	98 (27.6)	69 (26.4)	29 (51.8)	< 0.001
TMP/SMXR	53 (14.9)	39 (21.9)	14 (29.2)	0.337
COLIR	5 (1.4)	5 (1.7)	0 (0)	0.592
New definition*				
DTR	75 (21.1)	52 (17.7)	23 (37.1)	0.001
*Therapeutic management*				
ID consultation	148 (41.7)	119 (40.6)	29 (46.8)	0.397
Source control	131 (36.9)	111 (37.9)	20 (32.3)	0.470
Appropriate empirical therapy	125 (35.2)	111 (38.4)	14 (23.7)	0.037
Combination empirical therapy	55 (15.5)	42 (18.9)	13 (29.5)	0.152
2 In vitro active combination empirical therapy	16 (4.5)	16 (12.8)	0	0.223
Appropriate definitive therapy	262 (73.8)	222 (76.8)	40 (67.8)	0.184
Combination definitive therapy	115 (32.4)	89 (32)	26 (47.3)	0.031
2 In vitro active combination definitive therapy (with drugs)	58 (16.3)	48 (20.7)	10 (23.8)	0.682

*BL/BLIR* betalactam/betalactamase inhibitor resistance, *BSI* bloodstream infection, *COLIR* colistin resistance, *CR* carbapenem resistance, *CRE* carbapenem-resistant Enterobacteriaceae, *CVC* central venous catheter, *DTR* difficult-to-treat resistance, *ECR* extended-spectrum cephalosporin resistance, *FQR* fluoroquinolone resistance, *ICU* intensive care unit, *IQR* interquartile range, *ID* Consultation Infectious Disease Consultation, *MDR* multidrug-resistance, *SOFA* sequential organ failure assessment, *TMP/SMXR* trimethoprim/sulfamethoxazole resistance, *XDR* extensively drug-resistance

^*^Resistance categories were mutually exclusive while antibiotic class resistances and new definition (DTR) were not

**Fig. 4:**
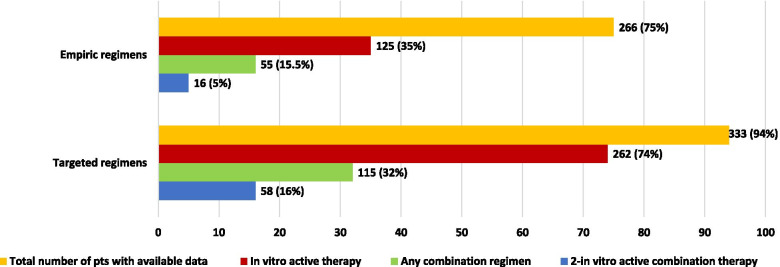
Antibiotic management of NFGN-BSI

As shown in Fig. 4, data on empirical and definitive antibiotic therapy were available for 266 (75%) and 333 (94%) patients, respectively. Active therapy was administered in 35.2% and 73.8% of empiric and definitive cohort patients, respectively. Empiric combination regimens were used in 55 (15.5%) patients with a 2-in vitro active drugs in 16 (4.5%). Combination regimens were used in 32.4% of patients in the definitive cohort, 16.3% with 2-in vitro active drugs. Empiric and definitive antibiotic regimens according to isolates are shown in Additional file 1: Tables S1 and S2.

All-cause 30-day mortality rate was 17.5%. Relapse at 90 days was observed in 7 patients (2%) within a median of 30 days (IQR 17–50) after index BCs. Compared to patients who were alive at day 30 (Table 2), non-surviving patients exhibited higher age, Charlson index and SOFA score, and higher rate of septic shock. Rates of *A. baumannii*, XDR or DTR isolation were significantly higher in non-surviving patients, while the rates of *P. aeruginosa* isolation and empirical active therapy were higher among survivors. For definitive therapy, the use of any combination was associated with higher mortality while that of 2-in vitro active combination did not. At multivariate analysis adjusted for DTR, *Stenotrophomonas maltophilia BSI*, *Acinetobacter baumanni BSI*, *Pseudomonas aeruginosa BSI*, active empiric therapy, active targeted therapy and source control, the independent risk factors for all-cause 30-day mortality were: age (odds ratio, OR 1.03, 95%, confidence interval, CI 1.00–1.04, p = 0.003), septic shock (OR 2.84, 95%CI 1.67–4.82, p < 0.001) and BSI due to *Acinetobacter baumannii* (OR 2.23, 95%CI 1.27–3.94, p = 0.005). Furthermore, risk factors for 30-day mortality have been estimated separately among patients with *P. aeruginosa* (Additional file 1: Table 3), *A. baumannii* (Additional file 1: Table 4) and *S. maltophilia* BSI (Additional file 1: Table 5).

## Discussion

In our large cohort of NFGN-BSI we have found high rates of DTR and carbapenem resistance, especially among *A. baumannii*. Empirical active therapy administration was significantly higher in surviving patients at univariate analysis, however it was not confirmed at multivariate analysis. Combination therapy, also with 2 active drug, was not associated to improving surviving at multivariate analysis. Of note, *A. baumannii* isolation resulted as independent risk factors for mortality at multivariate analysis. In our cohort nosocomial infections accounted for a large majority of cases (70%), mainly central venous catheter (CVC) related and pneumonia, according to literature [13, 14]. Therefore, promote and improve infection control programs would play a critical role in reducing the rates of this kind of nosocomial infections.

The increasing importance of the NFGN bacteria is also related to their complex antimicrobial resistance profile. In our cohort carbapenem resistance showed high prevalence, with 31.8% and 64.4% rates for *P. aeruginosa* and *A. baumannii* respectively. We have also analysed the prevalence of the new definition DTR. This definition reflects the use of second-line agents with poorer therapeutic index, resulting in a better prediction of poor outcome. In our cohort, the overall prevalence of DTR was 21.1%. It varied across species being highest among *A. baumannii* BSI with rates of 64.4%. This value was much higher than data present in literature [11, 15]. Similarly, DTR prevalence for *P.aeruginosa* BSI, accounted for 13.6%, higher than the rates showed previously [11, 15]. As expected, in *A. baumannii* strain, CR and DTR rates were comparable.

In our study active therapy did not result statistically associated with improved outcome as previously reported elsewhere. This finding deserves further investigation. Indeed, the classical way to define if an antimicrobial agent is useful to treat an infection is the MIC determination of strains. However, MIC determination have some concerns: (i) clinical laboratories cannot determine MICs with sufficient accuracy owing to the assay variation in the MIC test especially when automated or semi-automated methods are used, (ii) the MIC does not represent a concentration directly compared with in vivo concentration found during treatment; (iii) bacterial growth conditions in vitro could be different from those in vivo [16]. Also, the in vitro activity of antimicrobial often does not reflect the clinical feasibility due to the specific pharmacokinetic/toxicodynamic profile of the drugs and the source of infection [17]. These considerations could explain why in our cohort active therapy seems not associated to improving surviving. Similar experiences were previously reported elsewhere [18].

In our study, *A. baumannii* was an independent predictor of mortality. This is line with the characteristics of this pathogen that is commonly responsible for severe opportunistic nosocomial infections mainly in hospitalized immunocompromised patients [1–3]. Additionally, the complex antimicrobial resistance profile and the limited therapeutic arsenal for this strain may explain this result. In this scenario, polymyxins remains in vitro the most active agent. However, the in vitro activity of polymyxins not reflect the clinical feasibility due to the suboptimal pharmacokinetic/toxicodynamic profile of this class [17]. In all the study centers the polymyxin used for the treatment of *A. baumannii* BSI was colistin.

Our study has a number of limitations. Although we have analysed a large cohort of patients in three different centres, the results could be influenced by the epidemiology of a restricted area of our country. Also, our cohort is from all large tertiary teaching hospital reflecting the complexity and epidemiology of patients managed in similar institutions. The retrospective collection of patient and microbiological data could have limited integrity and accuracy. However, a senior investigator and three young investigators revised all clinical report forms (CRFs), and reconciled data reports and missing data with the medical records before including information in the database.

## Conclusions

To conclude, the overall prevalence of DTR was high in our NFGN BSI cohort, mainly among *Acinetobacter baumannii* episodes. Furthermore, *Acinetobacter baumannii* is showed to be an independent predictor of mortality. These evidences marked the urgent need of new therapeutic options against this pathogen.

## Supplementary Information

**Additional file 1: Table S1. **Empiric therapy according to isolates. **Table S2.** Definitive therapy according to isolates. **Table S3.** Univariable and multivariable analysis of risk factors for all-cause 30-day mortality in patients with *Pseudomonas aeruginosa* BSI. **Table S4.** Univariable and multivariable analysis of risk factors for all-cause 30-day mortality in patients with *Acinetobacter baumannii* BSI. **Table S5.** Univariable analysis of risk factors for all-cause 30-day mortality in patients with *Stenotrophomonas maltophilia* BSI*.

## Data Availability

The original data and materials from this study are available from the corresponding author on reasonable request.

## Abbreviations

BCs: Blood cultures
CDC: Centers for Disease Control and Prevention
CR: Carbapenem resistance
CRFs: Clinical report forms
CVC: Central venous catheter
DTR: Difficult to treat resistance
ESC: Extended-spectrum cephalosporins
EUCAST: Eeuropean Committee for Antimicrobial Susceptibility Testing
FQ: Fluoroquinolones
HIV: Human immunodeficiency virus
OR: Odds ratio
CI: Confidence interval
IQR: Interquartile range
MDR: Multi drug-resistant
MIC: Minimal inhibitory concentration
NFGN-BSI: Non-fermentative gram-negative bloodstream infection
PDR: Pan drug-resistant
SOFA score: Sequential organ failure assessment score
XDR: Extensively drug-resistant
