# Supplementary material for: Non-fermentative gram-negative bloodstream infection in northern Italy: a multicenter cohort study
Source: BMC Infect Dis. 2021 Aug 12;21:806. doi: 10.1186/s12879-021-06496-8 (PMC8359066; doi:10.1186/s12879-021-06496-8)
Supplement: Supplementary file 1 — Additional file 1: Table S1. Empiric therapy according to isolates. Table S2. Definitive therapy according to isolates. Table S3. Univariable and multivariable analysis of risk factors for all-cause 30-day mortality in patients with Pseudomonas aeruginosa BSI. Table S4. Univariable and multivariable analysis of risk factors for all-cause 30-day mortality in patients with Acinetobacter baumannii BSI. Table S5. Univariable analysis of risk factors for all-cause 30-day mortality in patients with Stenotrophomonas maltophilia BSI*. [file 12879_2021_6496_MOESM1_ESM.docx]

**ADDITIONAL DATA**

| **Table S1. Empiric therapy according to isolates** | | | | |
| --- | --- | --- | --- | --- |
|  | **Total number of patients with available data**  **N, (%)** | **In vitro Active Therapy**  **N, (%)** | **Combination therapy**  **N, (%)** | **2 -in vitro Active Combination Therapy N, (%)** |
| ***Pseudomonas aeruginosa*** | 196 (76) | 109 (55.6) | 43 (21.9) | 15 (7.6) |
| ***Acinetobacter baumannii*** | 41 (69.5) | 13 (31.7) | 8 (19.5) | 1 (2.4) |
| ***Stenotrophomonas maltophilia*** | 29 (76) | 3 (10.3) | 4 (13.8) | / |

| **Table S2. Definitive therapy according to isolates** | | | | |
| --- | --- | --- | --- | --- |
|  | **Total number of patients with available data**  **N, (%)** | **In vitro Active Therapy**  **N, (%)** | **Combination therapy**  **N, (%)** | **2 -in vitro Active Combination Therapy N, (%)** |
| ***Pseudomonas aeruginosa*** | 243 (94.2) | 208 (85.6) | 85 (35) | 48 (19.7) |
| ***Acinetobacter baumannii*** | 54 (91.5) | 37 (68.5) | 25 (46.3) | 6 (11.1) |
| ***Stenotrophomonas maltophilia*** | 36 (94.7) | 17 (47.2) | 5 (13.8) | 4 (11.1) |

| **Table S3. Univariable and multivariable analysis of risk factors for all-cause 30-day mortality in patients with *Pseudomonas aeruginosa* BSI** | | | | | | |
| --- | --- | --- | --- | --- | --- | --- |
|  |  | **Univariable analysis** | | | **Multivariable analysis*** | |
|  | **Total**  **N= 258 (%)** | **Survivors**  **N= 222 (%)** | **Non-survivors**  **N= 36 (%)** | ***p*** | **OR (95% IC)** | **P** |
| **Demographics** |  |  |  |  |  |  |
| Age (years) (median, IQR) | 68 (55-79) | 66 (54-78) | 75 (64-84) | 0.01 |  |  |
| Male sex | 172 (66.7) | 151 (68) | 21 (58.3) | 0.253 |  |  |
| **Comorbidities** |  |  |  |  |  |  |
| Charlson index (median, IQR) | 5 (4-7) | 5 (3-7) | 6 (5-8) | 0.017 |  |  |
| Immunosuppression | 69 (26.7) | 61 (27.5) | 8 (22.2) | 0.509 |  |  |
| **Ward of admission** |  |  |  | 0.567 |  |  |
| Medical | 53 (20.5) | 129 (58.1) | 23 (63.9) |  |  |  |
| ICU | 53 (20.5) | 45 (20.3) | 8 (22.8) |  |  |  |
| Surgical | 152 (58.9) | 45(20.3) | 5 (22.8) |  |  |  |
| **Site of BSI acquisition** |  |  |  | 0.357 |  |  |
| Community acquired | 60 (23.3) | 55 (24.8) | 5 (13.9) |  |  |  |
| Healthcare associated | 25 (9.7) | 21 (9.5) | 4 (11.4) |  |  |  |
| Hospital acquired | 173 (67.1) | 146 (65.8) | 27 (75) |  |  |  |
| CRE carrier at BSI onset | 35 (13.6) | 29 (13.1) | 6 (16.7) | 0.443 |  |  |
| **Clinical severity at BSI onset** |  |  |  |  |  |  |
| SOFA (median, IQR) | 3 (2-5) | 3 (2-5) | 4 (3-6) | 0.019 |  |  |
| Septic shock | 39 (15.1) | 28 (12.6) | 11 (30.6) | 0.005 |  |  |
| **Source of BSI** |  |  |  |  |  |  |
| Undefined | 138 (53.5) | 120 (54.1) | 18 (50) | 0.651 |  |  |
| CVC related | 69 (26.7) | 59 (26.6) | 10 (27.8) | 0.880 |  |  |
| Lower respiratory tract | 36 (14) | 29 (13.1) | 7 (19.4) | 0.305 |  |  |
| Biliary tract | 28 (10.9) | 24 (10.8) | 4 (11.1) | 0.957 |  |  |
| Urinary tract | 30 (11.6) | 26 (11.7) | 4 (11.1) | 0.917 |  |  |
| Intra-abdominal | 10 (3.9) | 10 (4.5) | 0 (0) | 0.194 |  |  |
| Complicated BSI | 24 (12.9) | 17 (12.8) | 7 (24.1) | 0.049 | 3.9 (1.27-10) | 0.03 |
| **Resistance phenotypes** |  |  |  | 0.014 |  |  |
| MDR | 15 (5.8) | 10 (4.5) | 5 (13.9) |  |  |  |
| XDR | 53 (20.5) | 42 (18.9) | 11 (30.6) |  |  |  |
| **Antibiotic class resistance** |  |  |  |  |  |  |
| ECR | 69 (26.7) | 55 (24.8) | 14 (38.9) | 0.076 |  |  |
| BL/BLIR | 75 (29.1) | 54 (24.3) | 21 (58.3) | <0.005 |  |  |
| CR | 82 (31.8) | 68 (30.6) | 14 (38.9) | 0.324 |  |  |
| FQR | 82 (31.8) | 63 (28.4) | 19 (52.8) | 0.004 |  |  |
| AminoglycosidesR | 58 (23.7) | 43 (20.4) | 15 (44.1) | 0.003 |  |  |
| TMP/SMXR | 18 (7) | 16 (7.2) | 2 (5.6) | 0.182 |  |  |
| COLIR | 3 (1.2) | 3 (1.4) | 0 (0) | 0.863 |  |  |
| **New definition** |  |  |  |  |  |  |
| DTR | 35 (13.6) | 28 (12.6) | 7 (19.4) | 0.267 |  |  |
| **Therapeutic management** |  |  |  |  |  |  |
| ID Consultation | 96 (37.2) | 80 (36) | 16 (44.4) | 0.333 |  |  |
| Source control | 90 (34.9) | 79 (35.6) | 11 (30.6) | 0.557 |  |  |
| Appropriate empirical therapy | 109 (55.6) | 100 (59.2) | 9 (33.3) | 0.012 | 0.25 (0.09-0.67) | 0.006 |
| Combination empirical therapy | 43 (21.9) | 35 (20.7) | 8 (29.6) | 0.298 |  |  |
| 2 *in vitro* active combination empirical therapy | 15 (7.6) | 15 (8.9) | 0 (0) | 0.107 |  |  |
| Appropriate definitive therapy | 208 (85.6) | 182 (86.3) | 26 (81.2) | 0.452 |  |  |
| Combination definitive therapy | 85 (35) | 69 (32.7) | 16 (50) | 0.056 |  |  |
| 2 *in vitro* active combination definitive therapy (with drugs) | 48 (19.8) | 39 (18.5) | 9 (28.1) | 0.202 |  |  |
| *Model adjusted for Age, Charlson Comorbidity Index, Septic shock, Resistance phenotypes (MDR, XDR), Combination definitive therapy. | | | | | | |

| **Table S4. Univariable and multivariable analysis of risk factors for all-cause 30-day mortality in patients with *Acinetobacter baumannii* BSI** | | | | | | |
| --- | --- | --- | --- | --- | --- | --- |
|  |  | ***Univariable analysis*** | | | ***Multivariable analysis**** | |
|  | **Total**  **N= 59 (%)** | **Survivors**  **N= 41 (%)** | **Non-survivors**  **N= 18 (%)** | ***p*** | ***OR* (95% IC)** | ***P*** |
| **Demographics** |  |  |  |  |  |  |
| Age (years) (median, IQR) | 67 (54-80) | 67 (53-78) | 71 (54-86) | 0.588 |  |  |
| Male sex | 40 (67.8) | 29 (70.7) | 11 (61.1) | 0.466 |  |  |
| **Comorbidities** |  |  |  |  |  |  |
| Charlson index (median, IQR) | 6 (3-8) | 6 (4-8) | 7 (3-9) | 0.100 |  |  |
| Immunosuppression | 10 (16.9) | 6 (14.6) | 4 (22.2) | 0.478 |  |  |
| **Ward of admission** |  |  |  | 0.250 |  |  |
| Medical | 32 (54.2) | 25 (61) | 7 (38.9) |  |  |  |
| ICU | 21 (35.6) | 13 (31.7) | 8 (44.4) |  |  |  |
| Surgical | 6 (10.2) | 3 (7.3) | 3 (16.7) |  |  |  |
| **Site of BSI acquisition** |  |  |  | 0.120 |  |  |
| Community acquired | 5 (8.5) | 5 (12.2) | 0 (0) |  |  |  |
| Healthcare associated | 5 (8.5) | 2 (4.9) | 3 (16.3) |  |  |  |
| Hospital acquired | 49 (83.1) | 34 (82.9) | 15 (83.3) |  |  |  |
| CRE carrier at BSI onset | 8 (13.6) | 5 (12.2) | 3 (16.7) | 0.474 |  |  |
| **Clinical severity at BSI onset** |  |  |  |  |  |  |
| SOFA (median, IQR) | 2 (1-5) | 2 (1-5) | 3 (2-5) | 0.211 |  |  |
| Septic shock | 14 (23.7) | 8 (19.5) | 6 (33.3) | 0.251 |  |  |
| **Source of BSI** |  |  |  |  |  |  |
| Undefined | 24 (40.7) | 14 (34.1) | 10 (55.6) | 0.123 |  |  |
| CVC related | 16 (27.1) | 11 (26.8) | 5 (27.8) | 0.940 |  |  |
| Lower respiratory tract | 9 (15.3) | 8 (19.5) | 1 (5.6) | 0.170 |  |  |
| Biliary tract | 10 (16.9) | 7 (17.1) | 3 (16.7) | 0.969 |  |  |
| Urinary tract | 1 (1.7) | 1 (2.4) | 0 (0) | 0.508 |  |  |
| Intra-abdominal | 5 (8.5) | 4 (9.8) | 1 (5.6) | 0.597 |  |  |
| Complicated BSI | 9 (20.5) | 6 (20) | 3 (21.4) | 0.913 |  |  |
| **Resistance phenotypes** |  |  |  |  |  |  |
| MDR | 19 (32.2) | 12 (29.3) | 7 (38.9) | 0.711 |  |  |
| XDR | 5 (8.5) | 4 (9.8) | 1 (5.6) | 0.947 |  |  |
| **Antibiotic class resistance** |  |  |  |  |  |  |
| CR | 41 (69.7) | 25 (61) | 16 (88.9) | 0.032 | 5.09 (1.01-25.6) | 0.048 |
| FQR | 37 (62.7) | 23 (56.1) | 14 (77.8) | 0.113 |  |  |
| TMP/SMXR | 33 (55.9) | 21 (51.2) | 12 (66.7) | 0.013 |  |  |
| COLIR | 1 (1.7) | 1 (2.4) | 0 (0) | 0.543 |  |  |
| **New definition** |  |  |  |  |  |  |
| DTR | 38 (64.4) | 25 (61) | 13 (72.7) | 0.406 |  |  |
| **Therapeutic management** |  |  |  |  |  |  |
| ID Consultation | 37 (62.7) | 27 (65.9) | 10 (55.6) | 0.451 |  |  |
| Source control | 21 (35.6) | 15 (36.6) | 6 (33.3) | 0.810 |  |  |
| Appropriate empirical therapy | 13 (31.7) | 10 (35.7) | 3 (23.1) | 0.418 |  |  |
| Combination empirical therapy | 8 (19.5) | 4 (14.3) | 4 (30.8) | 0.215 |  |  |
| 2 *in vitro* active combination empirical therapy | 1 (2.4) | 1 (3.6) | 0 (0) | 0.496 |  |  |
| Appropriate definitive therapy | 37 (68.5) | 29 (76.3) | 8 (50) | 0.057 |  |  |
| Combination definitive therapy | 25 (46.3) | 17 (44.7) | 8 (50) | 0.723 |  |  |
| 2 *in vitro* active combination definitive therapy (with drugs) | 6 (11.1) | 6 (15.8) | 0 (0) | 0.095 |  |  |
| * Model adjusted for Age, Appropriate definitive therapy, DTR. | | | | | | |

| **Table S5. Univariable analysis of risk factors for all-cause 30-day mortality in patients with *Stenotrophomonas maltophilia* BSI*** | | | | |
| --- | --- | --- | --- | --- |
|  | **Total**  **N= 38 (%)** | **Survivors**  **N= 30 (%)** | **Non-survivors**  **N= 8 (%)** | ***p*** |
| **Demographics** |  |  |  |  |
| Age (years) (median, IQR) | 67 (55-78) | 67 (51-77) | 67 (60-79) | 0.818 |
| Male sex | 21 (55.3) | 14 (46.7) | 7 (87.1) | 0.039 |
| **Comorbidities** |  |  |  |  |
| Charlson index (median, IQR) | 5 (3-8) | 5 (3-8) | 5 (3-9) | 0.950 |
| Immunosuppression | 8 (21.1) | 7 (23.3) | 1 (12.5) | 0.504 |
| **Ward of admission** |  |  |  | 0.166 |
| Medical | 26 (68.4) | 22 (73.3) | 4 (50) |  |
| ICU | 6 (15.8) | 3 (10) | 3 (37.5) |  |
| Surgical | 6 (15.8) | 5 (16.7) | 1 (12.5) |  |
| **Site of BSI acquisition** |  |  |  | 0.954 |
| Community acquired | 6 (15.8) | 5 (16.7) | 1 (12.5) |  |
| Healthcare associated | 5 (13.2) | 4 (13.3) | 1 (12.5) |  |
| Hospital acquired | 27 (71.1) | 21 (70) | 6 (75) |  |
| CRE carrier at BSI onset | 1 (2.6) | 1 (3.3) | 0 (0) | 0.067 |
| **Clinical severity at BSI onset** |  |  |  |  |
| SOFA (median, IQR) | 4 (3-6) | 3 (3-5) | 5 (5-6) | 0.070 |
| Septic shock | 6 (15.8) | 2 (6.7) | 4 (50) | 0.003 |
| **Source of BSI** |  |  |  |  |
| Undefined | 21 (55.3) | 16 (53.3) | 5 (62.5) | 0.643 |
| CVC related | 17 (44.7) | 16 (53.3) | 1 (12.5) | 0.042 |
| Lower respiratory tract | 4 (10.5) | 2 (6.7) | 2 (25) | 0.138 |
| Biliary tract | 3 (7.9) | 3 (10) | 0 | 0.358 |
| Intra-abdominal | 3 (7.9) | 2 (6.7) | 1 (12.5) | 0.592 |
| Complicated BSI | 5 (16.7) | 4 (18.1) | 1 (12.5) | 0.717 |
| **Antibiotic class resistance*** |  |  |  |  |
| FQR | 25 (65.8) | 21 (70) | 4 (50) | 0.289 |
| TMP/SMXR | 2 (5.3) | 2 (6.7) | 0 (0) | 0.199 |
| **New definition*** |  |  |  |  |
| DTR | 2 (5.3) | 2 (6.7) | 0 (0) | 0.459 |
| **Therapeutic management** |  |  |  |  |
| ID Consultation | 15 (39.5) | 12 (40) | 3 (37.5) | 0.889 |
| Source control | 20 (52.6) | 17 (56.7) | 3 (37.5) | 0.335 |
| Appropriate empirical therapy | 3 (10.3) | 1 (4) | 2 (50) | 0.006 |
| Combination empirical therapy | 4 (13.8) | 3 (12) | 1 (25) | 0.492 |
| Appropriate definitive therapy | 17 (47.2) | 11 (37.9) | 6 (85.7) | 0.023 |
| Combination definitive therapy | 5 (13.9) | 3 (10.3) | 2 (28.6) | 0.217 |
| 2 *in vitro* active combination definitive therapy (with drugs) | 4 (11.1) | 3 (10.3) | 1 (14.3) | 0.769 |
| * Multivariable analysis was not performed as the limited number of cases. | | | | |
